# A Critical Review of the Abilities, Determinants, and Possible Molecular Mechanisms of Seaweed Polysaccharides Antioxidants

**DOI:** 10.3390/ijms21207774

**Published:** 2020-10-21

**Authors:** Zhiwei Liu, Xian Sun

**Affiliations:** 1 School of Environment and Energy, South China University of Technology, Guangzhou 510006, China; zwliumost@126.com; 2 Guangdong Provincial Key Laboratory of Marine Resources and Coastal Engineering, School of Marine Sciences, Sun Yat-Sen University, Guangzhou 511458, China; 3 Zhuhai Key Laboratory of Marine Bioresources and Environment, School of Marine Sciences, Sun Yat-Sen University, Guangzhou 510275, China; 4 Southern Marine Science and Engineering Guangdong Laboratory (Zhuhai), Zhuhai 519080, China

**Keywords:** antioxidant, seaweed, polysaccharide, nuclear factor erythroid 2-related factor 2 (Nrf2)

## Abstract

Oxidative stress induces various cardiovascular, neurodegenerative, and cancer diseases, caused by excess reactive oxygen species (ROS). It is attributed to the lack of sufficient antioxidant defense capacity to eliminate unnecessary ROS. Seaweeds are largely cultivated for their edible and commercial purposes. Excessive proliferation of some seaweeds has occurred in coastal areas, causing environmental and economic disasters, and even threating human health. Removing and disposing of the excess seaweeds are costly and labor-intensive with few rewards. Therefore, improving the value of seaweeds utilizes this resource, but also deals with the accumulated biomass in the environment. Seaweed has been demonstrated to be a great source of polysaccharides antioxidants, which are effective in enhancing the antioxidant system in humans and animals. They have been reported to be a healthful method to prevent and/or reduce oxidative damage. Current studies indicate that they have a good potential for treating various diseases. Polysaccharides, the main components in seaweeds, are commonly used as industrial feedstock. They are readily extracted by aqueous and acetone solutions. This study attempts to review the current researches related to seaweed polysaccharides as an antioxidant. We discuss the main categories, their antioxidant abilities, their determinants, and their possible molecular mechanisms of action. This review proposes possible high-value ways to utilize seaweed resources.

## 1. Introduction

Reactive oxygen species (ROS) are byproducts of aerobic metabolism, mainly produced in the mitochondria. They contain free and non-free radical oxygen, such as hydrogen peroxide (H_2_O_2_), superoxide (O_2_^−^), singlet oxygen (1/2 O_2_), and hydroxyl radicals (∙OH). At high levels, ROS are toxic to cells as they impair the redox balance with high reactivity, resulting in damage to intracellular proteins, lipids, and nucleic acids. However, cells have evolved mechanisms to deal with ROS toxicity so that at low levels, they play an integral role in various cell signaling pathways as regulators, such as cytokine, transcription, neuro-modulation, immune-modulation, and apoptosis [1]. The balance between the formation of ROS and the ability to remove them is vital in biological systems, and a shift in the balance to ROS formation is termed “oxidative stress” [2], disordered redox signaling, and control [3]. Excess levels of ROS or the abnormal functioning of the antioxidant system, have been identified in various cardiovascular [4], pulmonary [5], diabetes [6], neurodegenerative [7], and cancer diseases [8]. Tightly controlling ROS levels is clearly crucial to biosystems. Both early and delayed mechanisms responding to increased ROS levels have evolved [9]. The early mechanism immediately removes the ROS through chemical reactions; the delayed mechanism is by gene expression of antioxidant enzymes and proteins that decrease oxidative damage [2,10]. More and more attention is being paid to preparing antioxidant compounds from nature in order to prevent and/or reduce oxidative damage.

Antioxidants have been deemed as substances with the potential at a relatively low concentration to delay or prevent the oxidation of a target substrate. They play the role of “free radical scavengers” by avoiding and repairing damage caused by oxidative stress. They enhance the immune system and decrease the risk of inflammation, cancer, aging, and hypertension [11,12,13]. Both endogenous and exogenous antioxidants counteract oxidative stress in eukaryotes. They are naturally internally produced or provided in foods and supplements [14]. Endogenous antioxidants are classified as either enzymatic or non-enzymatic [2]. Exogenous antioxidants play vital roles in promoting endogenous antioxidants, which cannot be produced and must be provided from foods or supplements such as vitamin E, vitamin C, carotenoids, trace metals (selenium, manganese, zinc), and flavonoids [15]. A deficiency of nutrient antioxidants is responsible for various chronic and degenerative pathologies and cancer [15].

While the benefits of antioxidants from terrestrial plants have been widely accepted, little attention has been focused on the same benefits from seaweed. Seaweed cultivation, which has been carried out for decades and is developing rapidly in the world, especially in China, has demonstrated effective remediation of contaminants and improvement in water quality [16,17,18,19,20,21]. Well-known species are *Laminaria*, *Pyropia*, *Gracilaria,* and *Undaria* [16], which are cultivated as industrial and edible resources. However, large-scale excessive proliferation of some seaweeds has occurred in coastal areas and is a widespread environmental problem. Examples include “green tides” caused by *Ulva* spp. along the coastal regions of the northern Yellow Sea, Jiangsu Province of China [22] and the eastern coast of Jeju Island, Korea [23], and “golden tides” initiated by *Sargassum* spp. along the Brazilian coast of the South Atlantic and the West Coast of Africa [24]. Mechanically harvesting the seaweeds in coastal or shore and then landfilling them is a common method to remove seaweeds; however, the process is costly with few rewards [25]. Thus, isolating and identifying useful compounds from seaweeds with novel applications that offer added economic value has become the focus of recent studies [26,27,28,29,30,31]. 

Seaweeds are rich in polysaccharides, proteins, vitamins, and minerals, as well as a great variety of secondary metabolites with diverse biological functions [32]. Due to their special structures, seaweed polysaccharides have been identified as being effective as antioxidants, immune-modulatory, anti-inflammatory, anti-coagulant, and anticancer agents [30,33,34,35,36]. For their health promoting effects, they are often recommended as food and food additives. Obviously, their potential in chemical and pharmaceutical industries is attractive. This study attempts to review the current research related to the antioxidant ability of seaweed polysaccharides, including the main categories, their antioxidant abilities, and determinants, as well as the possible molecular mechanisms. This review proposes possible high-value ways to utilize seaweeds. The keywords, “polysaccharide*”, “seaweed” or “marine algae”, and “antioxidant” were searched in “Web of Science” and “Scopus”, for the period between 2000 and 2020.

## 2. Polysaccharides from Seaweeds

Polysaccharides are the main components of seaweeds, widely used as industrial feedstock [29]. Various processes are used to extract polysaccharides from seaweeds, ultimately dissolving them into a liquid medium. Acidic or alkaline solutions traditionally facilitate extraction by interfering with hydrogen linkages between polysaccharides [37]. Novel methods have been applied in polysaccharides extraction, using fewer solvents, operating at lower temperatures, and decreasing extraction time [38]. Ultrasound assisted extraction (UAE) breaks down cells with frequencies higher than 20 kHz [39], microwave assisted extraction (MAE) disrupts hydrogen bonds, migrating dissolved ions with non-ionizing electromagnetic radiation of frequencies between 300 MHz and 300 GHz [40], and enzyme-assisted extraction (EAE) degrades cell walls [41]. However, no method is perfect, and yields may be low. In addition, the methods are not optimized and require adjustments to be boosted to an industrial scale [38].

The structural complexity of polysaccharides and their “unconventional” and heterogeneous sugar composition, sulfation, and other modifications limits their broader applications in the industry [38]. Polysaccharides have various brown, red, and green characteristics. Some important seaweed polysaccharides that have commercial value are fucoidan, alginates, and laminarin from brown algae; carrageenan and agar from red, and ulvan from green seaweeds [36,42,43,44,45,46,47].

### 2.1. Brown Seaweed Polysaccharides

Fucoidan, also called “fucan”, “fucosan”, or “sulfated fucan”, is composed of high percentages of L-fucose and sulfated ester groups. It is found in brown seaweeds and some marine invertebrates (such as sea urchins and sea cucumbers) [48,49]. Fucose, the preponderant constituent, is combined with other monomers, such as galactose, mannose, xylose, and residues of glucuronic acid [50,51]. Based on the backbone structure, fucoidans are divided into two subgroups: one is made up of α-1,3-L-fucopyranose and the other alternating 1,3-and 1,4-linked α-L-fucopyranose [52]. Fucoidan molecular weights range from 40 kDa to 1600 kDa. The amount of fucoidans in seaweeds varies with the seasons (highest during autumn), the species, and the development stage, from 0.1% to 20% of dry weight [53].

Alginates are primarily extracted from *Macrocystis pyrifera*, *Ascophyllum nodosum*, *Laminaria* spp., *Ecklonia maxima*, *Eisenia bicyclis*, *Lessonia nigricans,* and *Sargassum* spp. [54]. They are usually distributed in the cell walls as calcium, magnesium, and sodium salts and enhance the strength and flexibility of seaweed tissue [55]. The extraction process usually converts the cationic salt from the insoluble form to the soluble one, followed by eliminating impurities by successive dissolutions and precipitations [54]. Alginate is widely used in medical and pharmaceutical technology, and the cosmetic, food, agricultural, textile, and paper industries [56]. They are linear anionic unbranched copolymers, composed of β-1,4-D-mannuronic acid (M) and α-1,4-L-guluronic acid (G) and are usually described by their M/G ratio and average molecular weight, because their functions, physical properties, mechanical strength, and biocompatibility are largely dependent on those parameters [57].

Laminarin, a major carbohydrate reserve, is mainly composed of β-1,3-D-glucopyranose, with the β-1,6-linked D-glucopyranose units as branch-points or interchain residues [58]. Its solubility in water depends on the branching structure, as affected by interchain β-1,6-linkages. The antioxidant activity of laminarin has been linked to its molecular structure, degree, and length of branching and monosaccharide constituents [59]. According to the difference in terminal reducing end of the polymer chain, laminarin is divided into the G-type, which only contains glucopyranose, and the M-type with 1-O-linked D-mannitol [58,60]. The ratio of M-type to G-type varies by species, as high as 3:1 in *Laminaria digitate* [61] and absent in *Eisenia bicyclis*. The biological activities of laminarins vary according to species and are characterized by content, type (branchpoints or interchain), and the spatial distribution of the β-1,6-linkages [38].

### 2.2. Red Seaweed Polysaccharides

Carrageenan is a high molecular weight sulfated polysaccharide extracted from *Chondrus*, *Gigartina*, and various *Eucheuma* species from the red algal family, Rhodophyceae [62]. It is a major component of cell walls in red seaweeds and interacts with other bioactive compounds, such as proteins, lipids, and other polysaccharides [38]. It is composed of the base units, D-galactopyranosyl with one or two sulfate groups, linked via alternated (1→3)-β-_d_-and (1→4)-β-_d_-glucoside [36]. Depending on the number and position of the sulfate groups, carrageenans have been divided into 10 types, of which the kappa (κ), iota (ι), and lambda (λ) are of commercial significance. Carrageenan is edible and safe, largely used in food and pharmaceutical industries as a stabilizer, gelling agent, thickener, binder, and additive. However, there are reports that its consumption increases the risk of colitis [63,64]. In addition, in studies of carrageenan-induced paw edema and pleurisy and thrombosis in a tail thrombosis model, carrageenan is used to study the mechanisms involved in inflammation [65,66], antithrombosis, and thrombolysis [67,68] in laboratory animals, such as rats.

Agar is a mixture of gel polysaccharides, including agarose and agaropectin, uniquely found in the cell walls of some red seaweeds, specifically species in the families *Gracilariaceae*, *Gelidiaceae*, *Pterocladiaceae*, and *Gelidiellaceae* [69]. Agar is composed of alternating monomers, d-and l-galactose, linked via glycosidic bonds. Agarose, the major fraction of agar, making up about 70% of the polysaccharides, is composed of repeating d-galactose and 3,6-anhydro-l-galactose, with high molecular weight [70,71]. Compared to agarose, agaropectin has a lower molecular weight and higher amount of sulfate ester groups. The backbone of agaropectin is the same as agarose, substituted with various amounts of sulfate esters and d-glucuronic and pyruvic acids [69]. Agar, a phycocolloid from species in the genera *Gracilaria* and *Gelidium*, is broadly used in the food, pharmaceuticals, cosmetics, medical, and biotechnology industries [72].

### 2.3. Green Seaweed Polysaccharides

Ulvan is a uronic acid-rich sulfated polysaccharide, primarily composed of sulfated rhamnose, uronic acids (glucuronic acid and iduronic acid), and xylose, It is found in species mainly in the Ulvalean genera *Ulva* and *Enteromorpha* [73]. It is the major component of cell walls in green seaweed, occupying 9 to 36% of their dry weight [44]. The backbone of ulvan is mainly composed of repeating disaccharide units, α- and β-(1,4)-linked monosaccharides (rhamnose, xylose, glucuronic acid and iduronic acid). Ulvan has been reported to be effective as an immune modulator [74,75], and with antiviral [76,77] and anticoagulant properties [78,79]. It is regarded to have potential in nutraceutical, pharmaceutical, and cosmetic applications.

## 3. Antioxidant Ability of Polysaccharides

### 3.1. Radical Scavenging Capacity

The radical scavenging capacity of seaweed polysaccharides have been evaluated by two categories of assays: hydrogen atom transfer (HAT) and electron transfer (ET) reaction-based assays, depending on particular reactions [12]. Based on calculations of the ability of antioxidants to donate hydrogen, HAT-based methods are usually quick and independent of solvent and pH conditions [12,80]. In contrast, ET-based methods require relatively longer times, are typically expressed as percent decrease in product rather than kinetics [80,81]. Because of the dominant role of hydrogen atom transfer in biological redox reactions, HAT-based methods are considered to be more relevant to biology [12].

ET-based methods usually employ the scavenging capacity of 2,2-diphenyl-1-picrylhydrazyl (DPPH), the measurement of total phenolic content (TPC) by the Folin–Ciocalteu (FC) assay and the Ferric reducing antioxidant power (FRAP). The DPPH assay is simple and effective for evaluating the antioxidant capacity of extracts. It is based on the antioxidant potential to give a hydrogen atom to the synthetic nitrogen radical compound DPPH [82]. The Folin–Ciocalteu (FC) assay measures the reducing capacity of a sample by the Folin-Ciocalteu reagent (FCR), a mixture of phosphomolybdate and phosphotungstate [83]. The weight equivalents of standard antioxidants, such as gallic acid (GAE) [83], phloroglucinol (PGE) [84], and tannic acid equivalents (TAE), are used to measure the values of polyphenols in samples. Because of the similarity of the chemistry between FCR and an ET-based antioxidant capacity assay [12], good linear correlations have been frequently reported between the ‘‘total phenolic profiles’’ and ‘‘the antioxidant activity’’. However, the opposite results, i.e., a lack of correlation between TP content and antioxidant activity, have also been reported [85], suggesting that other components, such as chlorophyll and carotenoids, together with differences in the polyphenols profiles may affect the antioxidant activity [85]. The FRAP assay measures the power of the antioxidant to reduce Fe^3+^ to Fe^2+^ in acidic media (pH 3.6) maintained by 2,3,5-triphenyltetrazolium chloride (TPTZ). Due to the blue color of the ferrous (Fe^2+^) complex, antioxidant ability is calculated by the absorbance at 593 nm [86]. The equivalent of Fe^2+^ or reference standard antioxidants, such as Trolox, is expressed as the value of antioxidant ability. 

The assays of oxygen radical absorbance capacity (ORAC), 2,2’-azino-bis (3-ethylbenzothiazoline-6-sulphonic acid)(ABTS), superoxide anion (O_2_^−^), and hydroxyl (·OH) radicals scavenging activity are typically the HAT-based methods applied to measure the antioxidant ability of seaweed polysaccharides [80,87,88,89]. The ORAC assay measures the ability of the antioxidants to break radical chains by monitoring the inhibition of antioxidants to peroxyl radical induced oxidations [90]. Trolox is usually used as a standard antioxidant, and trolox equivalents (TE) are commensurately expressed as the ORAC values of the tested antioxidants [91]. Superoxide radical scavenging activity assay evaluates antioxidant capacities by supervising the inhibition in the photochemical reduction of nitro blue tetrazolium (NBT). The hydroxyl radical scavenging activity assay is based on the Fenton’s reaction [92]. Gallic acid is usually used as a positive control in the two assays [92,93].

Generally, more than two assays are applied to measure the antioxidant ability (Table 1). For example, the ulvan extracted from *U. pertusa* by the method of microwave-assisted extraction exerted a high antioxidant ability as evaluated by the radical-scavenging activity of DPPH and ABTS, and reducing power [94]. However, the results from different assays do not always agree, which may be attributed to the diverse mechanisms of the antioxidants. According to the action mode, there are primary and secondary antioxidants also referred to as chain breaking and preventive antioxidants [95]. Primary antioxidants commonly accept free radicals, breaking the propagation chain of autoxidation by inhibiting the initial step or interfering with the propagation step [96]. The activities of primary antioxidants are determined by their ability to donate hydrogen atoms to free radicals [97]. Secondary antioxidants alleviate oxidative stress by decreasing the rate of oxidation reactions via various mechanisms [98], such as providing H to a primary antioxidant, scavenging reactive oxygen and decomposing hydroperoxide [95]. Koh et al. reported that the antioxidant activity of *Undaria pinnatifida* fucoidan had a significantly less free radical scavenging ability than both the synthetic antioxidants, ascorbic acid, and BHA, but it had a hydroxyl radical scavenging activity similar to BHA although still lower than ascorbic acid [99]. Similar results were found with the *Sargassum binderi* fucoidan [100], indicating that fucoidan is a better secondary antioxidant than a primary one. However, Costa et al. measured the antioxidants of sulfated polysaccharides from 11 species of tropical marine seaweed, and found that only four species (*Caulerpa sertularioide*, *Dictyota cervicornis*, *Sargassum filipendula,* and *Dictyopteris delicatula*) showed hydroxyl radical scavenging activities, far less than gallic acid. Meanwhile, all species showed antioxidant ability through the assay of a total capacity antioxidant [93]. Oliveira reported a similar result of a sulfated galactan prepared from the red seaweed, *Gracilaria birdiae*, that showed no hydroxyl radical scavenging ability, suggesting that the antioxidant mechanism of seaweed sulfated polysaccharides is likely different [92].

### 3.2. Endogenous Antioxidant Ability

Besides their direct ROS scavenging ability, polysaccharides play a stronger role in the fight against oxidative stress by enhancing the endogenous antioxidant systems of humans and animals [44]. The endogenous antioxidant ability of polysaccharides is indirectly evaluated via the measurement of enzymatic (e.g., SOD, CAT, GPx) and oxidation products (such as malondialdehyde (MDA) and lipid peroxidation (LPO)) [44]. Table 2 and Table 3 summarized the recently reported enhancement of the endogenous antioxidant ability of seaweed polysaccharides according to cell lines and animal models, as related to diabetes [104], nephropathy [105], immunity [106], Alzheimer’s disease [42], pulmonary disease [107], and others. For examples, the neoagaro-oligosaccharides (NAOs), acid hydrolyzed from agar, have been reported to benefit the antioxidative system of type 2 diabetes mellitus (T2DM) mice by upregulating the activity of GPx and SOD while significantly reducing the concentration of MDA [108]. Ulvan alleviated the damage of RAW264.7 murine macrophage cell lines induced by H_2_O_2_ through the upregulation of levels of SOD and CAT [94]. Chen et al. investigated the ROS scavenging ability of agaro-oligosaccharides in vitro through the DPPH assay, then further studied the attenuating effect of oligosaccharides on ROS production in human liver L-02 cells treated by the oxidative agents, H_2_O_2_ and antimycin A [109]. However, agaro-oligosaccharides, such as agarobiose and agarotetraose, showed protection against oxidation in a concentration-dependent manner; they were also reported to induce oxidation at lower levels [109]. Additionally, pretreatment of polysaccharides before induction were applied in various studies with significantly protective effects, such as ABAP induced female Wistar rats [110], alcohol induced male Kunming mice [106], UVB radiation induced hairless Kun Ming mice [111], and H_2_O_2_-induced NT2 neurons [112]. Jin et al. compared four strategies of administering algal oligosaccharides (AOS) from *Gracilaria lemaneiformis* (Table 2), suggesting that taking AOS 2 h orally before alcohol consumption is the best strategy to protect the liver from alcohol damage [106]. A similar result was reported by Liu et al.—pretreatment of laminarin shows more significant changes in SOD, MDA, GSH, and CAT [107].

## 4. Determinants of Antioxidant Activity

The influence of the molecular weight of polysaccharides on antioxidant activity has been mentioned in various papers. The lower molecular weight sulphated polysaccharides tend to have a higher antioxidant activity. Lim et al. reported that among the fucoidans from *Fucus vesiculosus* degraded by gamma rays, via the assay method of ferric-reducing antioxidant power (FRAP), the lesser the molecular weight, the higher the antioxidant activity [122]. The fucoidan fraction with molecular weight lower than 10 kDa from *Undaria pinnatifida* showed a significantly higher secondary antioxidant activity than the crude fucoidan, the fraction with molecular weight cut off (MWCO) of 300 kDa, and even synthetic antioxidant butylated hydroxyanisole BHA [99]. Chen et al. reported that the fucoidan fractions with molecular weight between 5-10 k Da promoted the highest DPPH radical scavenging activity (48.3%) among fractions with molecular weight below 5 kDa, between 5–10 kDa, 10–30 kDa, 30–50 kDa, and over 50 kDa [123]. The stronger antioxidant activity of lower molecular weight polysaccharides may be attributed to their non-compact structure, which potentially makes more hydroxyl and amine groups available to neutralize free radicals [124]. However, agar oligosaccharides from *Gelidum amausii* showed an inverse consequence in which the oligosaccharide with higher molecular weight (2000–3800 Da) showed a higher antioxidation activity [125]. Sulfated polysaccharides from *Mastocarpus stellatus* showed similar results in which the fraction with the highest and lowest molecular weight were the best antioxidants, compared to the other fractions [124]. Therefore, other factors should be considered when evaluating the antioxidative ability of polysaccharides.

Sulfate content is also a vital factor affecting the antioxidant activity of fucoidan. There is a positive correlation between sulfate content and the scavenging superoxide radical ability in fucoidan from *Laminaria japonica* [126]. The ratio of sulfate content/fucose was proposed to be an effective indicator to evaluate the antioxidant activity of fucoidans [126]. Similar results were reported in the sulfated polysaccharides from the red seaweed *Mastocarpus stellatus* through the assay methods of FRAP-reducing power and ABTS-radical scavenging [124]. 

Other factors, such as the position of sulfate groups, and monosaccharide content and structure, influence the antioxidant activity of sulfated polysaccharides. However, Costa et al. analyzed the antioxidant ability of 11 seaweed polysaccharides, finding no correlation between sulfate content and superoxide anion scavenging ability [93]. Similar results were reported where concentration and structure were found to be the factors affecting the antioxidant ability of carrageenans rather than the degree of sulfation [47,102]. This indicates that not only sulfate content but also spatial patterns of sulfate groups determine the antioxidant activity of polysaccharides [47]. In *Ulva intestinalis*, alkaline-extracted sulfated polysaccharides exhibited no significantly higher DPPH radical scavenging ability, though they had higher sulfate content and lower molecular weight than water-extracted polysaccharides [127]. The study proposed that the antioxidant activity of the sulfated polysaccharides was more related to glucose content than sulfate content or molecular weight, which agreed with the conclusion by Lo et al. that the free-radical scavenging ability of polysaccharides were notably dependent on the monosaccharide composition [128]. Sokolova et al. measured the in vitro antioxidant properties of the various carrageenans (lambda-, iks-, kappa-, kappa/beta-and kappa/iota-) from *Gigartinaceae* and *Tichocarpaceae*, using the FRAP and PPM assays, and the radical scavenging of DPPH, superoxide anion, hydrogen peroxide, and nitric oxide [102]. Iks-carrageenan exhibited the most effective antioxidative ability, possibly because it has the highest content of sulfate groups and the 3,6-anhydrogalactose unit [102]. A similar result by Abad et al. reported that the antioxidant ability of κ-, ι- and λ-carrageenans followed the order of λ < ι < κ, according to the hydroxyl radical scavenging, reducing power, and DPPH radicals scavenging capacity assays [47].

Extraction and degradation methods affect the sulfate content at the same level of molecular weight, which determines the antioxidant activity of polysaccharides. Peasura et al. found that the antioxidant activities of sulphated polysaccharides from *Ulva intestinalis* were influenced by the extraction solvent (distilled water, 0.1 NHCl, and 0.1 N NaOH) and time (1, 3, 6, 12, and 24 h). The acid extract exhibited higher antioxidants than distilled water or alkali extract [127]. In *Fucus vesiculosus* fucoidans, the radical degradation method acquired almost 0.8-fold more sulfate content than acidic heating [122]. The degree of polymerization, an important factor affecting the antioxidant ability of carrageenans, depends on the methods of depolymerization. Sun et al. used four degradation methods, free radical depolymerization, mild acid hydrolysis, κ-carrageenase digestion and partial reductive hydrolysis, to degrade food-grade κ-carrageenan. Free radical depolymerization was proposed as an effective method to obtain hydrolysates with the highest antioxidant ability according to the structure analysis by ESI-MS and CID MS/MS and antioxidant activity assay [129]. It is attributed to the difference of the reducing sugar content, the degree of polymerization, and the carboxyl and sulfate groups affected by the methods of depolymerization [129]. Kang et al. explored the influence of the concentration of substrate and enzyme on the antioxidant activity of agaro-oligosaccharides degraded from agarose [103]. Using the assays of DPPH, ABTS, and FRAP, considerable antioxidant activities of agaro-oligosaccharides that depended on the degree of hydrolysis [103] were observed. Gamma irradiation also produced a low molecular weight laminarin through a random scission of chains, formatting more carbonyl groups and enhancing the antioxidation [59]. However, Rafiquzzaman et al. compared the antioxidant ability of carrageenans from *Hypnea musciformis* separately extracted by the conventional method and ultrasonic-assisted extraction (UAE). No significant difference was observed between the two methods [130].

## 5. Molecular Mechanism of Polysaccharide-Induced Antioxidant Ability

**Figure 1:**
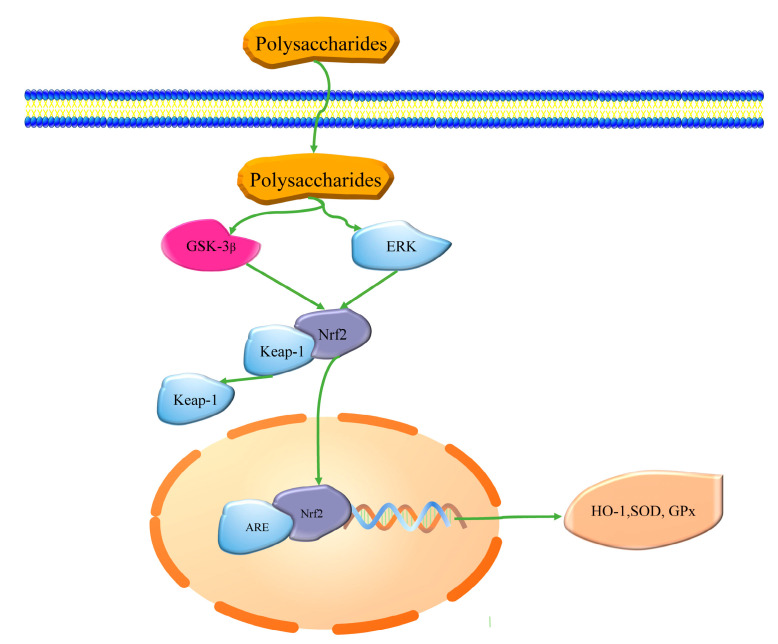
Schematic representation of Nrf2 signaling pathway regulated by seaweed polysaccharides.

**Figure 2:**
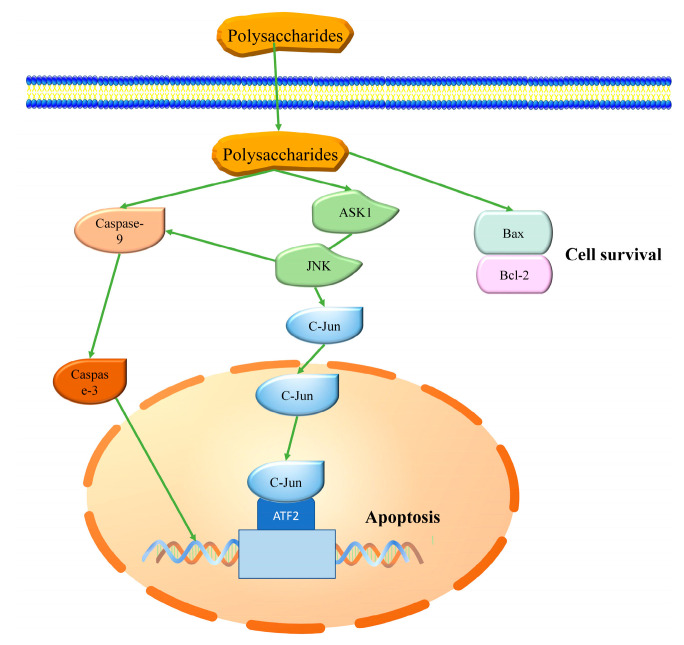
Schematic representation of apoptotic pathway regulated by seaweed polysaccharides.

A sequence of signaling cascades are available to eukaryotes to protect them from various harms and to maintain cellular redox homeostasis [131]. Besides immediately removing the generated ROS, antioxidants enhance the endogenous antioxidant system by upregulating the expression of genes encoding antioxidant enzymes and proteins to reduce the generation of noxious substances [2,10] (Table 4 and Figure 1). They also play roles in repairing the damage caused by ROS [132] which, when induced by oxidative stress, inhibits cell proliferation and causes apoptosis that is also regulated by a complex network of signaling pathways [133]. Antioxidants protect against oxidative stress damage induced by the regulation of apoptotic-related signaling pathways [134] (Table 4 and Figure 2).

### 5.1. Endogenous Antioxidant System

The activation of nuclear factor erythroid 2-related factor 2 (Nrf2)-driven antioxidant response element (ARE) is a crucial pathway in the response to oxidative stress. It induces various cytoprotective phase II enzymes [131,135,136]. The regulation of Nrf2 dependent on Kelch ECH associating protein 1 (Keap1) is the most characterized mechanism of ARE activation [137]. Nrf2 is an inducible cap ‘n’ collar type of transcription factor, normally degraded by the promotion of its repressor, Keap1, through the ubiquitin proteasome pathway [9,136]. When exposed to oxidant stress, the Keap1-Cul3 complex normally linked to Nrf2 is dissociated because of the modifications of Keap1 [10]. Nrf2 bound to ARE accumulates, promoting the production of the corresponding downstream phase II detoxifying enzymes and antioxidative proteins (e.g., superoxide dismutase-1 (SOD-1), hemeoxygenase-1 (HO-1), Glutamylcysteine synthetase (γ-GCS)) [9,138,139]. Ryu and Chung investigated the molecular mechanisms of fucoidan in the protection of human keratinocytes (HaCaT cells) from mild oxidative stress, indicating that the expression levels of HO-1, SOD-1, and Nrf2 are positively time-dependent following fucoidan treatment, whereas Keap1 expression was the opposite [140]. A similar positive effect of fucoidan treatment was also observed in the translocation of elevated Nrf2 from the cytosol into the nucleus, through the nuclear localization of Nrf2 by immunocytochemistry [140]. Fucoidan was also reported to extend the lifespan of the *Drosophila melanogaster* under heat stress by enhancement of the endogenous antioxidant system of upregulation of Nrf2 [141]. Positive regulation of Nrf2 by alginate was also reported in the H_2_O_2_-induced NT2 neural cell line by an increase in the levels of HO-1 and γ-GCS from upregulation of the expression of Nrf2 [112]. Laminarin also regulated NRF2 signaling pathways, suppressed KEAP1, and promoted NQO1, GCLC, and HO1 [108].

Upstream molecules of the Nrf2 signaling pathway regulated by seaweed polysaccharides, such as extracellular regulated protein kinases (ERK) [140] and glycogen synthase kinase3β (GSK-3β) [142], have been reported. The ERK/Nrf2 pathway regulates cellular protection against oxidative stress in various types of cells [143,144]. It was reported that fucoidan increased the level of ERK by positively upregulating the expression of Nrf2 [140]. Fucoidan also activates the Nrf2 signaling pathway by increasing the levels of GSK-3β in lipopolysaccharide (LPS)-induced male BALB/c mice [145].

### 5.2. Apoptotic Pathway

The Caspase and Bcl-2 families play important roles in apoptotic pathways [146]. Caspase-3 and Caspase-9, the former an effector and the latter an initiator, are crucial mediators of apoptosis [118,147]. Caspases are induced by an increase in intracellular ROS in response to oxidative damage [118]. Once Caspase-9 is activated, a cascade triggers the cleavage or activation of other downstream caspases such as caspase-3, causing cell death by apoptosis [132]. Fucoidans from *Dictyota mertensii* protect the pre-osteoblast-like cells (MC3T3-L1) from H_2_O_2_-induced apoptosis by decreasing intracellular ROS and depressing the activation of caspase-3 and caspase-9 [118]. Similar protections by polysaccharides, mainly fucoidans, depress the activation of caspase-3 and caspase-9 caused by inducers, such as FeSO_4_, CuSO_4_, ascorbate, and acetaminophen, and thereby ameliorate apoptosis [42,114,121,148]. 

Among ROS, H_2_O_2_ is a well-known apoptosis-inducing factor, which acts by several pathways [149]. The expression of the Bcl-2 family is regulated by H_2_O_2_ particularly by down-regulating anti-apoptotic Bcl-2 and increasing the expression of the pro-apoptotic Bcl-2- associated X protein (Bax). Bax plays a crucial role in mitochondria-dependent programed cell death. It induces necrotic cell death even without caspase activation in certain cases [150,151]. The ratio of Bax/Bcl has been demonstrated as a molecular control point in many apoptotic pathways and determines the progress of the cell death program [152]. Recent studies have shown that the decrease in activity of SOD, Gpx, and caspase-3, as well as the increased content of MDA and ratio of Bcl-2/Bax induced by Aβ-induced stress in Sprague–Dawley rats, were reversed by the fucoidan from *Laminaria japonica* [42]. Hong et al. also reported similar results in Sprague–Dawley rats, where fucoidan inhibited ROS accumulation and hepatic apoptosis was induced by acetaminophen with increasing activity of GSH, GPx, SOD, and expression of Bcl-2, as well as decreasing content of MDA and expression of Bax [114]. Polysaccharides from *Sargassum fulvellum* showed dose-dependent potential for scavenging intracellular ROS in 2,2-azobis (2-amidinopropane) hydrochloride (AAPH)-treated monkey kidney fibroblasts (Vero) cells. It inhibited apoptosis by downregulating Bax and caspase-3 and upregulating Bcl-xL and PARP [134]. 

Phosphorylation of c-Jun N-terminal protein kinases (JNKs) is another adverse consequence caused by oxidative stress, further aggravating oxidative stress in mitochondria, evoking dysfunction and DNA fragmentation, as well as cellular necrosis [113,153]. JNK activation upregulated the levels of Bax and promoted translocation to mitochondria in APAP hepatotoxicity [153]. Wang et al. showed that fucoidan markedly mitigated APPP-induced hepatotoxicity in the human normal hepatocyte HL-7702 cell line by alleviating mitochondria dysfunction, downregulation of a signal-regulating kinase 1(ASK1), followed by inhibition of JNK and Bax activation. Choi et al. also indicated that sulfated polysaccharide from *Hizikia fusiformis* effectively protected ethanol-induced cytotoxicity in IEC-6 cells by downregulation of JNK [119].

## 6. Conclusions

Polysaccharides are effective antioxidants both in vivo and in vitro. They are therapeutic for various diseases, such as diabetes, nephropathy, repressed immunity, Alzheimer’s, and pulmonary disease. The effects are determined by many factors, including molecular weight, sulfate content, position of sulfate groups, and monosaccharide content and structure. However, the determinants are complex and need further study. The exogenous antioxidants, polysaccharides, have been demonstrated to promote endogenous antioxidants such as CAT, SOD and GPx via upregulation of the expression of genes encoding antioxidant enzymes and proteins. The Nrf2 pathway is crucial to the oxidative stress response. Seaweed polysaccharides effectively upregulate Nrf2 by increasing the levels of Nrf2 upstream molecules such as ERK and GSK-3β, thus promoting the production of corresponding downstream Phase II detoxifying enzymes and antioxidative proteins such as SOD-1, HO-1, and γ-GCS. Seaweed polysaccharides also protect against oxidative stress by regulating apoptotic-related signaling pathways, such as Caspase-9, Bax and JNKs, in order to repair the damage caused by oxidative stress. We hope this review might be conducive to boost their value as antioxidants and facilitate seaweed polysaccharides as therapy for various diseases, in order to utilize seaweed resources to the utmost.

## Figures and Tables

**Table 1:** Summary of recently reported antioxidant ability of seaweed polysaccharides.

Type	Main Backbone	Source	Antioxidant Ability	Reference
Fucoidan	α-1,3-L-fucop yranose and the other alternating 1,3-and 1,4-linked α-L-fucopyranose	*Undaria pinnatifida* *from* New Zealand	At 2.0 mg/mL: DPPH (μg/mL TE): 7.43 ± 0.99; OH (%): 75.97 ± 1.69	[99]
		*Undaria pinnatifida* *from* Sigma-Aldrich	At 2.0 mg/mL: DPPH (μg/mL TE): 8.05 ± 1.49; ·OH (%): 75.32 ± 1.08	
		*Sargassum binderi* from Malaysia	At 2.0 mg/mL: TPC (mg GAE/100 g), 3.69 ± 0.15; DPPH (IC50 (mg/ml), 2.01 ± 0.29; O_2_^−^ (%), 26.78 ± 1.90; ·OH (%), 60.95 ± 0.69; FRAP (mg GAE/100 g), 0.60 ± 0.08	[100]
Alginates	β-1,4-D-mannuronic acid (M) and α-1,4-L-guluronic acid (G)	*Cystoseira barbata* from Tunisia	At 0.5 mg/L: DPPH (%), 74%; At 4 and 5 mg/ml, ·OH: 80 and 82%	[54]
		*Laminaria japonica* from China	MW of 1–6 KDa and 6–10 KDa: O_2_^−^ (I_50_): 8 μg mL^−1^ and 18 μg mL^−1^; OH (I_50_): 0.01 mg mL^−1^ and 0.03 mg mL^−1^	[57]
Laminarin	β-1,3-d-glucopyranose, with the β-1,6-linked d-glucopyranose units as branch-points or interchain residues	*Laminaria digitata*/*Ascophyllum nodosum* from SigmaeAldrich, USA	DPPH: reach 93.23%/87.57%; TPC (mg PGE/g): 0.343–0.365/0.110–0.166	[101]
Carrageenan	d-galactopyranosyl with one or two sulfate groups, linked via alternated (1→3)-β-d-and (1→4)-β-d-glucoside	*Chondrus armatus* and *C. pinnulatus* from Russian	At 1 mg/mL, 0.25–0.50 wt% substrate and 1.0–5.0 wt% enzyme: FRAP (mM AAE/g), 58.50–98.22; O_2_^−^ (%), 45.95–54.82%	[102]
Agar	repeating D-galactose and 3,6-anhydro-L-galactose	SigmaeAldrich, USA	At 10 mg/ml, DPPH (%), 16.47–22.71%; ABTS (%), 61.95–81.26%; FRAP, 0.95–1.46	[103]
Ulvan	repeating disaccharide units, α-and β-(1,4)-linked monosaccharides	*Ulva pertusa* from Korea	Among 0.025-0.800 mg/L: ABTS (%), 20.15–30.25; DPPH (%), 5.61–46.51;	[94]

AAE: ascorbic acid equivalents; ABTS: 2,2′-azino-bis (3-ethylbenzothiazoline-6-sulphonic acid radical scavenging activity; DPPH: 2,2-diphenyl-1-picrylhydrazyl radical scavenging activity; FRAP: Ferric reducing antioxidant power; GAE: gallic acid; MW: molecular weight; TE: trolox equivalent; TPC: total phenolic content; PGE: phloroglucinol equivalents; OH: hydroxyl radicals scavenging activity; O_2_^−^: Superoxide anion radicals scavenging activity.

**Table 2:** Summary of recently reported antioxidant ability from seaweed polysaccharides in animals.

Seaweed	Compound	Source	Administration	Dose (mg/kg)	Markers	Tissues	Model	Reference
Red	Carrageenan	*Kappaphycus alvarezii* from India/ Sigma-Aldrich, USA	2 days after induction, daily treatment, lasts 45 d	500, 750 and 1000	↑CAT, GPx, SOD, GST, and GSH; ↓LPO	Liver	Alloxan induced diabetic rats	[104]
	Sulfated polysaccharide	*Gracilaria* *Caudata* from Brazilian Atlantic coast	Before 18 h induction; pretreatment lasts 30 min	3 and 10	↑CAT and SOD		ABAP induced Female Wistar rats	[110]
	Oligosaccharide	*G. lemaneiformis*	Pretreatment 2 h before induction, once daily, lasts 21 d; pretreatment daily, lasts 2 weeks, followed by induction daily for 3 weeks; pretreatment immediately followed by induction, once daily, lasts 2 weeks; induction daily, lasts 3 weeks, followed by treatment daily for 2 weeks	50,150 and 250	↑GSH and SOD; ↓MDA		Alcohol induced male Kunming mice	[106]
Brown	Sulfated polysaccharide	*Sargassum fusiforme* from China	Pretreatment twice daily, lasts 5 days	5670	↑SOD; ↓MDA	Kidney	Contrast-induced nephropathy rats	[105]
			Pretreatment 2 h before induction, 3 times a week, lasts 9 weeks	200, 400, 600	↑SOD and CAT; ↓ROS and MDA	Skin	UVB radiation induced hairless Kun Ming mice	[111]
	Fucoidan	Cool Chemistry CO. Ltd., China	Pretreatment once daily, lasts 7 days	100 or 200	↑SOD, GSH and CAT ↓ROS and MDA	Liver	Acetaminophen induced male ICR mice	[113]
		*Laminaria* *Japonica* from China	After induction, treatment once daily, lasts 14 days	50, 100, 200	↑SDO and GPX; ↓MDA	Hippocampus	Aβ-induced Sprague–Dawley rats	[42]
		Sigma-Aldrich, USA	Pretreatment 2 h before induction, lasts 2 days	100	↑GSH, GPx and SOD; ↓MDA	liver	Acetaminophen induced Sprague–Dawleyrats	[114]
		*Saccharina japonica* from Ciyuan Biotechnology/*Sargassum horneri* from laboratory of Ningbo university	Twice daily, lasts 12 weeks	110, 220 and 440/30, 60, 120	↑CAT and SOD; ↓MDA	blood	yellow catfish (*Pelteobagrus fulvidraco*)	[115]
	Alginic acid oligosaccharide	Dalian Institute of Chemical Physics, Chinese Academy of Sciences	Daily, lasts 21 days	100	↑SOD, CAT, T-AOC and GPx; ↓MDA		weaned pigs	[116]
Green	Sulfated polysaccharide	*Ulva lactuca* from Mandapam region	After induction, treatment daily, lasts 4 weeks; pretreatment 4 weeks before induction	100	↑CAT and SOD	liver	D-galactosamine induced Adult male Albino Wistar rats	[117]

ABAP: 2,2′-azobis (2-methylpropionamidine) dihydrochloride; Aβ: amyloid beta peptide; CAT: Catalase; GPx: glutathione peroxidase; GSH: glutathione; GST: glutathione S-transferase; ICR: Institute of Cancer Research; LPO: Lipid peroxidation; MDA: malondialdehyde; SOD: superoxide dismutase; TAOC: total antioxidant capacity; UVB: ultraviolet B; The arrows upward represent increase and the downward represent decrease.

**Table 3:** Summary of recently reported antioxidant ability from seaweed polysaccharides in cell lines.

Seaweed	Compound	Source	Administration	Dose (mg/mL)	Markers	Model	Reference
Brown	Alginate	Sigma Aldrich, USA	Pretreatment 1 h before induction;	0.030	↑GSH	H_2_O_2_-induced NT2 neurons	[112]
	Fucoidan	Cool Chemistry CO., China	Pretreatment 4 h before induction	0.025. 0.050 and 0.100	↑GSH and SOD; ↓ROS and MDA	Acetaminophen induced HL-7702 cell line	[113]
		*Dictyota mertensii* from Brazil	Co-treatment with induction, lasts 6 h	0.050-0.500	↑SOD; ↓ROS,	H_2_O_2_ induced pre- osteoblast-like cells (MC3T3-L1)	[118]
	Laminarin	Sigma-Aldrich, USA	Treatment 1 h before or after induction	0.020	↑SOD, GSH and CAT; ↓MDA	H_2_O_2_ induced Human lung fibroblasts MRC-5 cells	[107]
	Sulfated polysaccharide	*Hizikia fusiformis* from Korean	Pretreatment 24 h before induction	0.500	↑GSH	Ethanol induced rat intestinal cell line IEC-6	[119]
Green	Ulvan	*Ulva pertusa* from Korea	Pretreatment 2 h before induction	0.100 and 0.200	↑SOD and CAT	H_2_O_2_ induced RAW264.7 murine macrophage cell line	[94]
	Sulfated Polysaccharides	*Monostroma nitidum* from Korea	Treatment after induction, lasts 24 h	0.050, 0.100 and 0.200	↑SOD	Lipid-loaded HepG2 cells	[120]
		*Udotea flabellum* from Brazil	Co-treatment, with induction, lasts 1.5 h	1.000	↑SOD and GSH; ↓MDA	FeSO4 or CuSO4 and ascorbate induced 3T3 fibroblasts	[121]

CAT: Catalase; GPx: glutathione peroxidase; GSH: glutathione; MDA: malondialdehyde; SOD: superoxide dismutase; The arrows upward represent increase and the downward represent decrease.

**Table 4:** Summary of recently reported molecular mechanisms of seaweed polysaccharides against oxidative stress.

Seaweed	Compound	Source	Administration	Dose	Model	Possible Mechanism	Reference
Brown	Sulfated polysaccharide	*Hizikia fusiformis*	Pretreatment 24 h before induction	0.5 mg/L	Ethanol induced rat intestinal cell line IEC-6	↓JNK phosphorylation	[119]
	Fucoidan	*Dictyota mertensii* from Brazil	Co-treatment with induction, lasts for 6 h	0.5 mg/L	H_2_O_2_ induced pre- osteoblast-like cells (MC3T3-L1)	↓caspase-3 and caspase-9	[118]
		*Sargassum fusiforme* from China	Co-treatment for 50 days	400, 800 and 1600 mg/L	Heat Stress induced *Drosophila melanogaster*	↑*Nrf2*; ↓*keap1*	[141]
		*Laminaria* *Japonica* from China	After induction, treatment once daily, lasts for 14 days	100 and 200 mg/Kg	Aβ induced Sprague–Dawley rats	↑Bcl-2/Bax; ↓caspase-3	[42]
		Sigma-Aldrich, USA	24 h pretreatment	0.1 and 1 μM	Aβ induced rat cholinergic basal forebrain neurons	↓caspase-3 and caspase-9	[148]
			24 h treatment	30 mg/L	human keratinocyte cell line (HaCaT)	↑HO-1, SOD-1, Nrf2 and ERK; ↓Keap1	[140]
			Pretreatment 1 h before induction	20, 40, and 80 mg/kg	Lipopolysaccharide (LPS)-induced male BALB/c mice	↑GSK-3β, Nrf2, and HO-1	[145]
			Pretreatment 2 h before induction, lasts 2 days	100 mg/kg	Acetaminophen induced Sprague–Dawley rats	↑Bcl-2; ↓Bax and caspse-3	[114]
	Alginate	Sigma Aldrich, USA	Pretreatment 1 h before induction	30 mg/L	H_2_O_2_ induced NT2 neural cell line	↑HO-1, γ-GCS, Hsp-70 and Nrf2; ↓caspase-3 and NF-κB	[112]
	Laminarin	Sigma-Aldrich, USA	Treatment 1 h before or after induction, lasts 24 h	20 mg/L	H_2_O_2_ induced Human lung fibroblasts MRC-5 cells	↑Nrf2, NQO1, GCLC and HO1; ↓KEAP1	[107]
	Fucoidan	Cool Chemistry CO.	Pretreatment once daily, lasts 7 days	100 and 200 mg/kg	APAP included human normal hepatocyte HL-7702 cell line	↑Nrf2; ↓JNK Phosphorylation and ASK1	[113]
Green	sulfated polysaccharide	*Udotea flabellum*	Co-treatment, with induction, lasts 1.5 h	1000 mg/L	FeSO_4_ or CuSO_4_ and ascorbate induced 3T3 fibroblasts	↓caspase-3 and caspase-9	[121]

ASK1: apoptosis signal-regulating kinase 1; Bax: Bcl-2 associated X protein; Bcl-2: B-cell lymphoma 2; ERK: extracellular regulated protein kinases; GSK-3β: Glycogen synthase kinase3β; HO-1: hemeoxygenase-1; JNK: jun N-terminal kinase; Keap1: Kelch ECH associating protein 1; Nrf2: nuclear factor erythroid 2-related factor 2; γ-GCS: Glutamylcysteine synthetase; The arrows upward represent increase and the downward represent decrease.

## References

[1] Mittler R., Vanderauwera S., Suzuki N., Miller G., Tognetti V.B., Vandepoele K., Gollery M., Shulaev V., Van Breusegem F. (2011). ROS signaling: The new wave?. Trends Plant Sci..

[2] Gupta R.K., Patel A.K., Shah N., Choudhary A.K., Jha U.K., Yadav U.C., Gupta P.K., Pakuwal U. (2014). Oxidative Stress and Antioxidants in Disease and Cancer: A Review. Asian Pac. J. Cancer Prev..

[3] Jones D.P. (2006). Redefining Oxidative Stress. Antioxid. Redox Signal..

[4] Faria A., Persaud S.J. (2017). Cardiac oxidative stress in diabetes: Mechanisms and therapeutic potential. Pharmacol. Ther..

[5] Wang D., Li H., Weir E.K., Xu Y., Xu D., Chen Y. (2019). Dimethylarginine dimethylaminohydrolase 1 deficiency aggravates monocrotaline-induced pulmonary oxidative stress, pulmonary arterial hypertension and right heart failure in rats. Int. J. Cardiol..

[6] Domingueti C.P., Dusse L.M.S., Carvalho M.d.G., de Sousa L.P., Gomes K.B., Fernandes A.P. (2016). Diabetes mellitus: The linkage between oxidative stress, inflammation, hypercoagulability and vascular complications. J. Diabetes Complicat..

[7] Singh A., Kukreti R., Saso L., Kukreti S. (2019). Oxidative Stress: A Key Modulator in Neurodegenerative Diseases. Molecules.

[8] Saha S.K., Lee S.B., Won J., Choi H.Y., Kim K., Yang G.-M., Dayem A.A., Cho S. (2017). Correlation between Oxidative Stress, Nutrition, and Cancer Initiation. Int. J. Mol. Sci..

[9] Kim K.C., Hyun Y.J., Hewage S.R.K.M., Piao M.J., Kang K.A., Kang H.K., Koh Y.S., Ahn M.J., Hyun J.W. (2017). 3-Bromo-4,5-dihydroxybenzaldehyde enhances the level of reduced glutathione via the Nrf2-mediated pathway in human keratinocytes. Mar. Drugs.

[10] Poljsak B., Šuput D., Milisav I. (2013). Achieving the Balance between ROS and Antioxidants: When to Use the Synthetic Antioxidants. Oxid. Med. Cell. Longev..

[11] Abdollahi M., Moridani M.Y., Aruoma O.I., Mostafalou S. (2014). Oxidative Stress in Aging. Oxid. Med. Cell. Longev..

[12] Tierney M.S., Croft A.K., Hayes M. (2010). A review of antihypertensive and antioxidant activities in macroalgae. Bot. Mar..

[13] Yang S., Lian G. (2020). ROS and diseases: Role in metabolism and energy supply. Mol. Cell. Biochem..

[14] Espinosa-Diez C. (2015). Antioxidant responses and cellular adjustments to oxidative stress. Redox Biol..

[15] Gupta R.K., Singh N. (2013). Morinda citrifolia (Noni) Alters Oxidative Stress Marker and Antioxidant Activity in Cervical Cancer Cell Lines. Asian Pac. J. Cancer Prev..

[16] Yang Y., Chai Z., Wang Q., Chen W., He Z., Jiang S. (2015). Cultivation of seaweed *Gracilaria* in Chinese coastal waters and its contribution to environmental improvements. Algal Res..

[17] Luo H., Wang Q., Nie X., Ren H., Shen Z., Xie X., Yang Y. (2018). Heavy Metal Contamination in the Cultivated Oyster *Crassostrea rivularis* and Associated Health Risks from a Typical Mariculture Zone in the South China Sea. Bull. Environ. Contam. Toxicol..

[18] Sun X., Liu Z., Jiang Q., Yang Y. (2019). Concentrations of various elements in seaweed and seawater from Shen’ao Bay, Nan’ao Island, Guangdong coast, China: Environmental monitoring and the bioremediation potential of the seaweed. Sci. Total Environ..

[19] Chen B., Xia J., Zou D., Zhang X. (2019). Responses to ocean acidification and diurnal temperature variation in a commercially farmed seaweed, *Pyropia haitanensis* (Rhodophyta). Eur. J. Phycol..

[20] Chen B., Lin L., Ma Z., Zhang T., Chen W., Zou D. (2019). Carbon and nitrogen accumulation and interspecific competition in two algae species, *Pyropia haitanensis* and *Ulva lactuca*, under ocean acidification conditions. Aquac. Int..

[21] Zou D., Gao K. (2010). Acquisition of inorganic carbon by *Endarachne binghamiae* (Scytosiphonales, Phaeophyceae). Eur. J. Phycol..

[22] Zhang C., Lu J., Wu J., Luo Y. (2017). Removal of phenanthrene from coastal waters by green tide algae *Ulva prolifera*. Sci. Total Environ..

[23] Cho H.-M., Kim G., Shin K.-H. (2019). Tracing nitrogen sources fueling coastal green tides off a volcanic island using radon and nitrogen isotopic tracers. Sci. Total Environ..

[24] Li J., Zhu Y., Wang C., Wei W., Liu Z., Tian Y., Zong P., Qiao Y., Qin S. (2020). Golden seaweed tides from beach inundations as a valuable sustainable fuel resource: Fast pyrolysis characteristics, product distribution and pathway study on *Sargassum horneri* based on model compounds. Algal Res. Biomass Biofuels Bioprod..

[25] Resiere D., Valentino R., Nevière R., Banydeen R., Gueye P., Florentin J., Cabié A., Lebrun T., Mégarbane B., Guerrier G. (2018). *Sargassum* seaweed on Caribbean islands: An international public health concern. Lancet.

[26] Cornish M.L., Critchley A.T., Mouritsen O.G. (2015). A role for dietary macroalgae in the amelioration of certain risk factors associated with cardiovascular disease. Phycologia.

[27] Gammone M.A., D’Orazio N. (2015). Anti-obesity activity of the marine carotenoid fucoxanthin. Mar. Drugs.

[28] Rathnayake A.U., Abuine R., Kim Y.-J., Byun H.-G. (2019). Anti-Alzheimer’s Materials Isolated from Marine Bio-resources: A Review. Curr. Alzheimer Res..

[29] Park E.-J., Pezzuto J.M. (2013). Antioxidant Marine Products in Cancer Chemoprevention. Antioxid. Redox Signal..

[30] Sanjeewa K.K.A., Kang N., Ahn G., Jee Y., Kim Y.-T., Jeon Y.-J. (2018). Bioactive potentials of sulfated polysaccharides isolated from brown seaweed *Sargassum* spp in related to human health applications: A review. Food Hydrocoll..

[31] Niu T., Fu G., Zhou J., Han H., Chen J., Wu W., Chen H. (2020). Floridoside Exhibits Antioxidant Properties by Activating HO-1 Expression via p38/ERK MAPK Pathway. Mar. Drugs.

[32] Francisco J., Horta A., Pedrosa R., Afonso C., Cardoso C., Bandarra N.M., Gil M.M. (2020). Bioaccessibility of antioxidants and fatty acids from *Fucus spiralis*. Foods.

[33] Cardoso S.M., Pereira O.R., Seca A.M.L., Pinto D.C.G.A., Silva A.M.S. (2015). Seaweeds as Preventive Agents for Cardiovascular Diseases: From Nutrients to Functional Foods. Mar. Drugs.

[34] Phull A.R., Kim S.J. (2017). Fucoidan as bio-functional molecule: Insights into the anti-inflammatory potential and associated molecular mechanisms. J. Funct. Foods.

[35] Pinteus S., Lemos M.F.L., Alves C., Neugebauer A., Silva J., Thomas O.P., Botana L.M., Gaspar H., Pedrosa R. (2018). Marine invasive macroalgae: Turning a real threat into a major opportunity—The biotechnological potential of *Sargassum muticum* and *Asparagopsis armata*. Algal Res. Biomass Biofuels Bioprod..

[36] Liu Z., Gao T., Yang Y., Meng F., Zhan F., Jiang Q., Sun X. (2019). Anti-Cancer Activity of Porphyran and Carrageenan from Red Seaweeds. Molecules.

[37] Abdul Khalil H.P.S., Lai T.K., Tye Y.Y., Rizal S., Chong E.W.N., Yap S.W., Hamzah A.A., Nurul Fazita M.R., Paridah M.T. (2018). A review of extractions of seaweed hydrocolloids: Properties and applications. Express Polym. Lett..

[38] Jönsson M., Allahgholi L., Sardari R.R.R., Hreggviðsson G.O., Nordberg Karlsson E. (2020). Extraction and Modification of Macroalgal Polysaccharides for Current and Next-Generation Applications. Molecules.

[39] Mtetwa M.D., Qian L.S., Zhu H.A., Cui F.J., Yang Y. (2020). Ultrasound-assisted extraction and antioxidant activity of polysaccharides from Acanthus ilicifolius. J. Food Meas. Charact..

[40] Yuan Y., Macquarrie D. (2015). Microwave assisted extraction of sulfated polysaccharides (fucoidan) from Ascophyllum nodosum and its antioxidant activity. Carbohydr. Polym..

[41] Vásquez V., Martínez R., Bernal C. (2019). Enzyme-assisted extraction of proteins from the seaweeds *Macrocystis pyrifera* and *Chondracanthus chamissoi*: Characterization of the extracts and their bioactive potential. J. Appl. Phycol..

[42] Gao Y., Li C., Yin J., Shen J., Wang H., Wu Y., Jin H. (2012). Fucoidan, a sulfated polysaccharide from brown algae, improves cognitive impairment induced by infusion of A beta peptide in rats. Environ. Toxicol. Pharmacol..

[43] Ahn J.H., Kim D.W., Park C.W., Kim B., Sim H., Kim H.S., Lee T.-K., Lee J.-C., Yang G.E., Her Y. (2020). Laminarin Attenuates Ultraviolet-Induced Skin Damage by Reducing Superoxide Anion Levels and Increasing Endogenous Antioxidants in the Dorsal Skin of Mice. Mar. Drugs.

[44] Kidgell J.T., Magnusson M., de Nys R., Glasson C.R.K. (2019). Ulvan: A systematic review of extraction, composition and function. Algal Res..

[45] Fernando I.P.S., Kim K.-N., Kim D., Jeon Y.-J. (2019). Algal polysaccharides: Potential bioactive substances for cosmeceutical applications. Crit. Rev. Biotechnol..

[46] Falkeborg M., Cheong L.-Z., Gianfico C., Sztukiel K.M., Kristensen K., Glasius M., Xu X., Guo Z. (2014). Alginate oligosaccharides: Enzymatic preparation and antioxidant property evaluation. Food Chem..

[47] Abad L.V., Relleve L.S., Racadio C.D.T., Aranilla C.T., De la Rosa A.M. (2013). Antioxidant activity potential of gamma irradiated carrageenan. Appl. Radiat. Isot..

[48] Bilan M.I., Grachev A.A., Ustuzhanina N.E., Shashkov A.S., Usov A.I. (2002). Structure of a fucoidan from the brown seaweed *Fucus evanescens* C.Ag. Carbohydr. Res..

[49] Li B., Lu F., Wei X., Zhao R. (2008). Fucoidan: Structure and bioactivity. Molecules.

[50] Ale M.T., Mikkelsen J.D., Meyer A.S. (2011). Important Determinants for Fucoidan Bioactivity: A Critical Review of Structure-Function Relations and Extraction Methods for Fucose-Containing Sulfated Polysaccharides from Brown Seaweeds. Mar. Drugs.

[51] Lim S.J., Mustapha W.A.W., Maskat M.Y., Latip J., Badri K.H., Hassan O. (2016). Chemical properties and toxicology studies of fucoidan extracted from Malaysian *Sargassum binderi*. Food Sci. Biotechnol..

[52] Jiao G., Yu G., Zhang J., Ewart H.S. (2011). Chemical structures and bioactivities of sulfated polysaccharides from marine algae. Mar. Drugs.

[53] Fletcher H.R., Biller P., Ross A.B., Adams J.M.M. (2017). The seasonal variation of fucoidan within three species of brown macroalgae. Algal Res..

[54] Sellimi S., Younes I., Ayed H.B., Maalej H., Montero V., Rinaudo M., Dahia M., Mechichi T., Hajji M., Nasri M. (2015). Structural, physicochemical and antioxidant properties of sodium alginate isolated from a Tunisian brown seaweed. Int. J. Biol. Macromol..

[55] Draget K.I., Smidsrød O., Skjåk-Bræk G. (2005). Alginates from Algae. Biopolym. Online.

[56] Brownlee I.A., Seal C.J., Wilcox M., Dettmar P.W., Pearson J.P. (2009). Applications of Alginates in Food. Alginates: Biology and Applications.

[57] Zhao X., Li B., Xue C., Sun L. (2012). Effect of molecular weight on the antioxidant property of low molecular weight alginate from *Laminaria japonica*. J. Appl. Phycol..

[58] Graiff A., Ruth W., Kragl U., Karsten U. (2016). Chemical characterization and quantification of the brown algal storage compound laminarin—A new methodological approach. J. Appl. Phycol..

[59] Choi J.I., Kim H.J., Lee J.W. (2011). Structural feature and antioxidant activity of low molecular weight laminarin degraded by gamma irradiation. Food Chem..

[60] Kadam S.U., Tiwari B.K., O’Donnell C.P. (2015). Extraction, structure and biofunctional activities of laminarin from brown algae. Int. J. Food Sci. Technol..

[61] Read S.M., Currie G., Bacic A. (1996). Analysis of the structural heterogeneity of laminarin by electrospray-ionisation-mass spectrometry. Carbohydr. Res..

[62] Yu X., Zhou C., Yang H., Huang X., Ma H., Qin X., Hu J. (2015). Effect of ultrasonic treatment on the degradation and inhibition cancer cell lines of polysaccharides from *Porphyra yezoensis*. Carbohydr. Polym..

[63] Necas J., Bartosikova L. (2013). Carrageenan: A review. Veterinární Medicína.

[64] Shang Q., Sun W., Shan X., Jiang H., Yu G. (2017). Carrageenan-induced colitis is associated with decreased population of anti-inflammatory bacterium, *Akkermansia muciniphila*, in the gut microbiota of C57BL/6J mice. Toxicol. Lett..

[65] Ghorbanzadeh B., Mansouri M., Hemmati A., Naghizadeh B., Mard S., Rezaie A. (2015). A study of the mechanisms underlying the anti-inflammatory effect of ellagic acid in carrageenan-induced paw edema in rats. Indian J. Pharmacol..

[66] Venkatranganna M.V., Bhonde R.R., Shree N., Venkategowda S. (2017). Treatment with adipose derived mesenchymal stem cells and their conditioned media reverse carrageenan induced paw oedema in db/db mice. Biomed. Pharmacother..

[67] Arslan R., Bektas N., Bor Z., Sener E. (2015). Evaluation of the antithrombotic effects of *Crataegus monogyna* and *Crataegus davisii* in the carrageenan-induced tail thrombosis model. Pharm. Biol..

[68] Zhang Y.-L., Xi M.-Z., Choi Y.-B., Lee B.-H. (2017). Antithrombotic Effect of Fermented Ophiopogon japonicus in Thrombosis-Induced Rat Models. J. Med. Food.

[69] Lee W.-K., Lim Y.-Y., Leow A.T.-C., Namasivayam P., Abdullah J.O., Ho C.-L. (2017). Factors affecting yield and gelling properties of agar. J. Appl. Phycol..

[70] Lee W.-K., Lim Y.-Y., Leow A.T.-C., Namasivayam P., Abdullah J.O., Ho C.-L. (2017). Biosynthesis of agar in red seaweeds: A review. Carbohydr. Polym..

[71] María P., Elena F., Herminia D. (2016). Antimicrobial Action of Compounds from Marine Seaweed. Mar. Drugs.

[72] Bixler H.J., Porse H. (2011). A decade of change in the seaweed hydrocolloids industry. J. Appl. Phycol..

[73] Lahaye M., Robic A. (2007). Structure and functional properties of Ulvan, a polysaccharide from green seaweeds. Biomacromolecules.

[74] Fernández-Díaz C., Coste O., Malta E.-J. (2017). Polymer chitosan nanoparticles functionalized with *Ulva ohnoi* extracts boost in vitro ulvan immunostimulant effect in *Solea senegalensis* macrophages. Algal Res..

[75] Ponce M., Zuasti E., Anguís V., Fernández-Díaz C. (2020). Effects of the sulfated polysaccharide ulvan from *Ulva ohnoi* on the modulation of the immune response in Senegalese sole (*Solea senegalensis*). Fish Shellfish Immunol..

[76] Adrien A., Bonnet A., Dufour D., Baudouin S., Maugard T., Bridiau N. (2017). Pilot production of ulvans from *Ulva* sp. and their effects on hyaluronan and collagen production in cultured dermal fibroblasts. Carbohydr. Polym..

[77] Aguilar-Briseño J.A., Cruz-Suarez L.E., Sassi J.F., Ricque-Marie D., Trejo-Avila L.M. (2015). Sulphated Polysaccharides from *Ulva clathrata* and *Cladosiphon okamuranus* Seaweeds both Inhibit Viral Attachment/Entry and Cell-Cell Fusion, in NDV Infection. Mar. Drugs.

[78] Peso-Echarri P., Frontela-Saseta C., Gonzalez-Bermudez C.A. (2012). Polysaccharides from seaweed as ingredients in marine aquaculture feeding: Alginate, carrageenan and ulvan. Rev. Biol. Mar. Oceanogr..

[79] Qi H., Liu X., Wang K., Liu D., Huang L., Liu S., Zhang Q. (2013). Subchronic toxicity study of ulvan from *Ulva pertusa* (Chlorophyta) in Wistar rats. Food Chem. Toxicol..

[80] Shahidi F., Zhong Y. (2015). Measurement of antioxidant activity. J. Funct. Foods.

[81] Prior R.L., Wu X., Schaich K. (2005). Standardized methods for the determination of antioxidant capacity and phenolics in foods and dietary supplements. J. Agric. Food Chem..

[82] Stratil P., Klejdus B., Kubáň V. (2006). Determination of total content of phenolic compounds and their antioxidant activity in vegetables–evaluation of spectrophotometric methods. J. Agric. Food Chem..

[83] Rico D., Alonso de Linaje A., Herrero A., Asensio-Vegas C., Miranda J., Martínez-Villaluenga C., de Luis D.A., Martin-Diana A.B. (2018). Carob by-products and seaweeds for the development of functional bread. J. Food Process. Preserv..

[84] Olate-Gallegos C., Barriga A., Vergara C., Fredes C., García P., Giménez B., Robert P. (2019). Identification of Polyphenols from Chilean Brown Seaweeds Extracts by LC-DAD-ESI-MS/MS. J. Aquat. Food Prod. Technol..

[85] Belda M., Sanchez D., Bover E., Prieto B., Padron C., Cejalvo D., Miguel Lloris J. (2016). Extraction of polyphenols in *Himanthalia elongata* and determination by high performance liquid chromatography with diode array detector prior to its potential use against oxidative stress. J. Chromatogr. B Anal. Technol. Biomed. Life Sci..

[86] Benitez Garcia I., Duenas Ledezma A.K., Martinez Montano E., Salazar Leyva J.A., Carrera E., Osuna Ruiz I. (2020). Identification and Quantification of Plant Growth Regulators and Antioxidant Compounds in Aqueous Extracts of *Padina durvillaei* and *Ulva lactuca*. Agronomy.

[87] Barahona T., Chandia N.P., Encinas M.V., Matsuhiro B., Zuniga E.A. (2011). Antioxidant capacity of sulfated polysaccharides from seaweeds. A kinetic approach. Food Hydrocoll..

[88] Cui C., Lu J., Sun-Waterhouse D., Mu L., Sun W., Zhao M., Zhao H. (2016). Polysaccharides from *Laminaria japonica*: Structural characteristics and antioxidant activity. LWT Food Sci. Technol..

[89] Lorbeer A.J., Charoensiddhi S., Lahnstein J., Lars C., Franco C.M.M., Bulone V., Zhang W. (2017). Sequential extraction and characterization of fucoidans and alginates from *Ecklonia radiata*, *Macrocystis pyrifera*, *Durvillaea potatorum*, and *Seirococcus axillaris*. J. Appl. Phycol..

[90] Uribe E., Vega-Gálvez A., García V., Pastén A., López J., Goñi G. (2019). Effect of different drying methods on phytochemical content and amino acid and fatty acid profiles of the green seaweed, *Ulva* spp.. J. Appl. Phycol..

[91] Agregán R., Lorenzo J.M., Munekata P.E.S., Dominguez R., Carballo J., Franco D. (2017). Assessment of the antioxidant activity of *Bifurcaria bifurcata* aqueous extract on canola oil. Effect of extract concentration on the oxidation stability and volatile compound generation during oil storage. Food Res. Int..

[92] Oliveira L.C.B.P., Queiroz M.F., Fidelis G.P., Melo K.R.T., Rocha H.A.O. (2020). Antioxidant Sulfated Polysaccharide from Edible Red Seaweed *Gracilaria birdiae* Is an Inhibitor of Calcium Oxalate Crystal Formation. Molecules.

[93] Costa L.S., Fidelis G.P., Cordeiro S.L., Oliveira R.M., Sabry D.A., Câmara R.B.G., Nobre L.T.D.B., Costa M.S.S.P., Almeida-Lima J., Farias E.H.C. (2010). Biological activities of sulfated polysaccharides from tropical seaweeds. Biomed. Pharmacother..

[94] Le B., Golokhvast K.S., Yang S.H., Sun S. (2019). Optimization of Microwave-Assisted Extraction of Polysaccharides from *Ulva pertusa* and Evaluation of Their Antioxidant Activity. Antioxidants.

[95] Senanayake S.P.J.N., Wanasundara P.K.J.P.D., Shahidi F. (2020). Antioxidants: Science, Technology, and Applications. Bailey’s Industrial Oil and Fat Products.

[96] Daramola B., Adegoke G. (2011). Bitter kola (*Garcinia kola*) seeds and health management potential. Nuts and Seeds in Health and Disease Prevention.

[97] Berdahl D., Nahas R., Barren J. (2010). Synthetic and natural antioxidant additives in food stabilization: Current applications and future research. Oxidation in Foods and Beverages and Antioxidant Applications.

[98] Mishra R., Bisht S.S. (2011). Antioxidants and their characterization. J. Pharm. Res..

[99] Koh H.S.A., Lu J., Zhou W. (2019). Structure characterization and antioxidant activity of fucoidan isolated from *Undaria pinnatifida* grown in New Zealand. Carbohydr. Polym..

[100] Lim S.J., Wan Aida W.M., Maskat M.Y., Mamot S., Ropien J., Mazita Mohd D. (2014). Isolation and antioxidant capacity of fucoidan from selected Malaysian seaweeds. Food Hydrocoll..

[101] Kadam S.U., O’Donnell C.P., Rai D.K., Hossain M.B., Burgess C.M., Walsh D., Tiwari B.K. (2015). Laminarin from Irish brown seaweeds *Ascophyllum nodosum* and *Laminaria hyperborea*: Ultrasound assisted extraction, characterization and bioactivity. Mar. Drugs.

[102] Sokolova E.V., Barabanova A.O., Bogdanovich R.N., Khomenko V.A., Solov’eva T.F., Yermak I.M. (2011). In vitro antioxidant properties of red algal polysaccharides. Biomed. Prev. Nutr..

[103] Kang O.L., Ghani M., Hassan O., Rahmati S., Ramli N. (2014). Novel agaro-oligosaccharide production through enzymatic hydrolysis: Physicochemical properties and antioxidant activities. Food Hydrocoll..

[104] Sanjivkumar M., Chandran M.N., Suganya A.M., Immanuel G. (2020). Investigation on bio-properties and in-vivo antioxidant potential of carrageenans against alloxan induced oxidative stress in Wistar albino rats. Int. J. Biol. Macromol..

[105] Dai M., Zhou Y.-L., Jiang T., Luo C.-D., Wang H., Du W., Wang M. (2019). Characterization of Polysaccharides Extracted from *Sargassum fusiforme* and Its Effective Prevention of Contrast-Induced Nephropathy via Enhancing Antioxidant Capacity. Int. J. Polym. Sci..

[106] Jin M., Liu H., Hou Y., Chan Z., Di W., Li L., Zeng R. (2017). Preparation, characterization and alcoholic liver injury protective effects of algal oligosaccharides from *Gracilaria lemaneiformis*. Food Res. Int..

[107] Liu X., Liu H., Zhai Y., Li Y., Zhu X., Zhang W. (2017). Laminarin protects against hydrogen peroxide-induced oxidative damage in MRC-5 cells possibly via regulating NRF2. PeerJ.

[108] Lin F., Yang D., Huang Y., Zhao Y., Ye J., Xiao M. (2019). The Potential of Neoagaro-Oligosaccharides as a Treatment of Type II Diabetes in Mice. Mar. Drugs.

[109] Chen H.M., Yan X.J. (2005). Antioxidant activities of agaro-oligosaccharides with different degrees of polymerization in cell-based system. Biochim. Biophys. Acta.

[110] Cavalcante Alencar P.O., Lima G.C., Barros F.C.N., Costa L.E.C., Ribeiro C.V.P.E., Sousa W.M., Sombra V.G., Abreu C.M.W.S., Abreu E.S., Pontes E.O.B. (2019). A novel antioxidant sulfated polysaccharide from the algae *Gracilaria caudata*: In vitro and in vivo activities. Food Hydrocoll..

[111] Ye Y., Ji D., You L., Zhou L., Zhao Z., Brennan C. (2018). Structural properties and protective effect of *Sargassum fusiforme* polysaccharides against ultraviolet B radiation in hairless Kun Ming mice. J. Funct. Foods.

[112] Eftekharzadeh B., Khodagholi F., Abdi A., Maghsoudi N. (2010). Alginate protects NT2 neurons against H_2_O_2_-induced neurotoxicity. Carbohydr. Polym..

[113] Wang Y.-Q., Wei J.-G., Tu M.-J., Gu J.-G., Zhang W. (2018). Fucoidan alleviates acetaminophen-induced hepatotoxicity via oxidative stress inhibition and Nrf2 translocation. Int. J. Mol. Sci..

[114] Hong S.-W., Lee H.-S., Jung K.H., Lee H., Hong S.-S. (2012). Protective effect of fucoidan against acetaminophen-induced liver injury. Arch. Pharm. Res..

[115] Yang Q., Yang R., Li M., Zhou Q., Liang X., Elmada Z.C. (2014). Effects of dietary fucoidan on the blood constituents, anti-oxidation and innate immunity of juvenile yellow catfish (*Pelteobagrus fulvidraco*). Fish Shellfish Immunol..

[116] Wan J., Jiang F., Xu Q., Chen D., He J. (2016). Alginic acid oligosaccharide accelerates weaned pig growth through regulating antioxidant capacity, immunity and intestinal development. RSC Adv..

[117] Sathivel A., Balavinayagamani, Rao B.R.H., Devaki T. (2014). Sulfated polysaccharide isolated from *Ulva lactuca* attenuates D-galactosamine induced DNA fragmentation and necrosis during liver damage in rats. Pharm. Biol..

[118] Fidelis G.P., Ferreira Silva C.H., Duarte Barreto Nobre L.T., Medeiros V.P., Oliveira Rocha H.A., Costa L.S. (2019). Antioxidant Fucoidans Obtained from Tropical Seaweed Protect Pre-Osteoblastic Cells from Hydrogen Peroxide-Induced Damage. Mar. Drugs.

[119] Choi E.-Y., Hwang H.-J., Nam T.-J. (2010). Protective effect of a polysaccharide from *Hizikia fusiformis* against ethanol-induced cytotoxicity in IEC-6 cells. Toxicol. In Vitro.

[120] Hoang M.H., Kim J.-Y., Lee J.H., You S.G., Lee S.-J. (2015). Antioxidative, hypolipidemic, and anti-inflammatory activities of sulfated polysaccharides from *Monostroma nitidum*. Food Sci. Biotechnol..

[121] Presa F.B., Mendes Marques M.L., Silva Viana R.L., Duarte Barreto Nobre L.T., Costa L.S., Oliveira Rocha H.A. (2018). The Protective Role of Sulfated Polysaccharides from Green Seaweed *Udotea flabellum* in Cells Exposed to Oxidative Damage. Mar. Drugs.

[122] Lim S., Choi J., Park H. (2015). Antioxidant activities of fucoidan degraded by gamma irradiation and acidic hydrolysis. Radiat. Phys. Chem..

[123] Chen Q., Kou L., Wang F., Wang Y. (2019). Size-dependent whitening activity of enzyme-degraded fucoidan from *Laminaria japonica*. Carbohydr. Polym..

[124] Gómez-Ordóñez E., Jiménez-Escrig A., Rupérez P. (2014). Bioactivity of sulfated polysaccharides from the edible red seaweed *Mastocarpus stellatus*. Bioact. Carbohydr. Diet. Fibre.

[125] Wang J., Jiang X., Mou H., Guan H. (2004). Anti-oxidation of agar oligosaccharides produced by agarase from a marine bacterium. J. Appl. Phycol..

[126] Wang J., Zhang Q., Zhang Z., Li Z. (2008). Antioxidant activity of sulfated polysaccharide fractions extracted from *Laminaria japonica*. Int. J. Biol. Macromol..

[127] Peasura N., Laohakunjit N., Kerdchoechuen O., Wanlapa S. (2015). Characteristics and antioxidant of *Ulva intestinalis* sulphated polysaccharides extracted with different solvents. Int. J. Biol. Macromol..

[128] Lo T.C.-T., Chang C.A., Chiu K.-H., Tsay P.-K., Jen J.-F. (2011). Correlation evaluation of antioxidant properties on the monosaccharide components and glycosyl linkages of polysaccharide with different measuring methods. Carbohydr. Polym..

[129] Sun Y., Yang B., Wu Y., Liu Y., Gu X., Zhang H., Wang C., Cao H., Huang L., Wang Z. (2015). Structural characterization and antioxidant activities of kappa-carrageenan oligosaccharides degraded by different methods. Food Chem..

[130] Rafiquzzaman S.M., Ahmed R., Lee J.M., Noh G., Jo G., Kong I.-S. (2016). Improved methods for isolation of carrageenan from *Hypnea musciformis* and its antioxidant activity. J. Appl. Phycol..

[131] Dinkova-Kostova A.T., Holtzclaw W.D., Kensler T.W. (2005). The Role of Keap1 in Cellular Protective Responses. Chem. Res. Toxicol..

[132] Juarez-Portilla C., Olivares-Banuelos T., Molina-Jimenez T., Armando Sanchez-Salcedo J., Del Moral D.I., Meza-Menchaca T., Flores-Munoz M., Lopez-Franco O., Roldan-Roldan G., Ortega A. (2019). Seaweeds-derived compounds modulating effects on signal transduction pathways: A systematic review. Phytomedicine.

[133] Hwang J.Y., Park J.H., Kim M.J., Kim W.J., Ha K.-T., Choi B.T., Lee S.-Y., Shin H.K. (2019). Isolinderalactone regulates the BCL-2/caspase-3/PARP pathway and suppresses tumor growth in a human glioblastoma multiforme xenograft mouse model. Cancer Lett..

[134] Wang L., Oh J.Y., Hwang J., Ko J.Y., Jeon Y.-J., Ryu B. (2019). In Vitro and In Vivo Antioxidant Activities of Polysaccharides Isolated from Celluclast-Assisted Extract of an Edible Brown Seaweed, Sargassum fulvellum. Antioxidants.

[135] Baird L., Dinkova-Kostova A.T. (2011). The cytoprotective role of the Keap1–Nrf2 pathway. Arch. Toxicol..

[136] Nabavi S.F., Barber A.J., Spagnuolo C., Russo G.L., Daglia M., Nabavi S.M., Sobarzo-Sánchez E. (2016). Nrf2 as molecular target for polyphenols: A novel therapeutic strategy in diabetic retinopathy. Crit. Rev. Clin. Lab. Sci..

[137] Ma Q. (2013). Role of Nrf2 in Oxidative Stress and Toxicity. Annu. Rev. Pharmacol. Toxicol..

[138] Kim E.-Y., Choi Y.H., Nam T.-J. (2018). Identification and antioxidant activity of synthetic peptides from phycobiliproteins of *Pyropia yezoensis*. Int. J. Mol. Med..

[139] Wang R., Paul V.J., Luesch H. (2013). Seaweed extracts and unsaturated fatty acid constituents from the green alga *Ulva lactuca* as activators of the cytoprotective Nrf2-ARE pathway. Free Radic. Biol. Med..

[140] Ryu M.J., Chung H.S. (2016). Fucoidan reduces oxidative stress by regulating the gene expression of HO-1 and SOD-1 through the Nrf2/ERK signaling pathway in HaCaT cells. Mol. Med. Rep..

[141] Zhang Y., Xu M., Hu C., Liu A., Chen J., Gu C., Zhang X., You C., Tong H., Wu M. (2019). *Sargassum fusiforme* Fucoidan SP2 Extends the Lifespan of Drosophila melanogaster by Upregulating the Nrf2-Mediated Antioxidant Signaling Pathway. Oxid. Med. Cell. Longev..

[142] Jiang W., Luo T., Li S., Zhou Y., Shen X.Y., He F., Xu J., Wang H.Q., Ken A. (2016). Quercetin Protects against Okadaic Acid-Induced Injury via MAPK and PI3K/Akt/GSK3β Signaling Pathways in HT22 Hippocampal Neurons. PLoS ONE.

[143] Li X., Darzynkiewicz Z. (2000). Cleavage of Poly (ADP-ribose) polymerase measured in situ in individual cells: Relationship to DNA fragmentation and cell cycle position during apoptosis. Exp. Cell Res..

[144] Yu R., Chen C., Mo Y.Y., Hebbar V., Owuor E.D., Tan T.H., Kong A.N.T. (2000). Activation of Mitogen-activated Protein Kinase Pathways Induces Antioxidant Response Element-mediated Gene Expression via a Nrf2-dependent Mechanism. J. Biol. Chem..

[145] Zhu D.-Z., Wang Y.-T., Zhuo Y.-L., Zhu K.-J., Wang X.-Z., Liu A.-J. (2020). Fucoidan inhibits LPS-induced acute lung injury in mice through regulating GSK-3 beta-Nrf2 signaling pathway. Arch. Pharm. Res..

[146] Ding X., Ge B., Wang M., Zhou H., Sang R., Yu Y., Xu L., Zhang X. (2020). *Inonotus obliquus* polysaccharide ameliorates impaired reproductive function caused by *Toxoplasma gondii* infection in male mice via regulating Nrf2-PI3K/AKT pathway. Int. J. Biol. Macromol..

[147] Kim B., Srivastava S.K., Kim S.-H. (2015). Caspase-9 as a therapeutic target for treating cancer. Expert Opin. Ther. Targets.

[148] Jhamandas J.H., Wie M.B., Harris K., MacTavish D., Kar S. (2005). Fucoidan inhibits cellular and neurotoxic effects of β-amyloid (Aβ) in rat cholinergic basal forebrain neurons. Eur. J. Neurosci..

[149] Faucher K., Rabinovitch-Chable H., Cook-Moreau J., Barriere G., Sturtz F., Rigaud M. (2005). Overexpression of human GPX1 modifies Bax to Bcl-2 apoptotic ratio in human endothelial cells. Mol. Cell. Biochem..

[150] Hardwick J.M., Soane L. (2013). Multiple Functions of BCL-2 Family Proteins. Cold Spring Harb. Perspect. Biol..

[151] Wang S., Sorenson C.M., Sheibani N. (2005). Attenuation of retinal vascular development and neovascularization during oxygen-induced ischemic retinopathy in Bcl-2−/− mice. Dev. Biol..

[152] Yang Q., Wang S., Xie Y., Sun J., Wang J. (2010). HPLC analysis of Ganoderma lucidum polysaccharides and its effect on antioxidant enzymes activity and Bax, Bcl-2 expression. Int. J. Biol. Macromol..

[153] Gunawan B.K., Liu Z.-X., Han D., Hanawa N., Gaarde W.A., Kaplowitz N. (2006). c-Jun N-Terminal Kinase Plays a Major Role in Murine Acetaminophen Hepatotoxicity. Gastroenterology.

